# Culinary Nutrition Programming for Members of a Community-Based Cancer Program

**DOI:** 10.3390/nu18050858

**Published:** 2026-03-06

**Authors:** Billie Jane C. Hermosura, Meaghan E. Kavanagh, Jaime Slavin, David J. A. Jenkins, Amy Symington

**Affiliations:** 1 Faculty of Education, University of Ottawa, Ottawa, ON K1N 9A8, Canada; bhermosu@uottawa.ca; 2 Department of Nutrition, Harvard T.H. Chan School of Public Health, Boston, MA 02115, USA; mkavanagh@hsph.harvard.edu; 3 Department of Nutritional Sciences, Temerty Faculty of Medicine, University of Toronto, Toronto, ON M5S 1A8, Canada; david.jenkins@utoronto.ca; 4 Independent Researcher, Toronto, ON M5G 1N8, Canada; jaime@jaimeslavin.com; 5 Department of Medicine, Temerty Faculty of Medicine, University of Toronto, Toronto, ON M5S 1A8, Canada; 6 Division of Endocrinology and Metabolism, St. Michael’s Hospital, Toronto, ON M5B 1A6, Canada; 7 Li Ka Shing Knowledge Institute, St. Michael’s Hospital, Toronto, ON M5B 1A6, Canada

**Keywords:** cancer survivorship, culinary, teaching kitchens, food literacy, nutrition, plant-based diets, food attitudes, dietary behavior, mindful eating, multidisciplinary care

## Abstract

(1) Background: Nutrition research in cancer care has largely focused on disease prevention and management, overlooking the importance of food literacy. Culinary cancer care programs may address this gap by facilitating the practical application of nutrition through culinary skills, fostering social connections over nutrient-dense meals, and supporting individuals during periods of physical and social vulnerability. The Not-Just-Supper Club (NJSC) at Gilda’s Club Toronto (GT) is a community-based culinary cancer care program delivering evidence-based, plant-forward meals. The objectives of this study were to examine how NJSC supports its members and to provide recommendations to inform future models of culinary cancer care programs. (2) Methods: An explanatory sequential mixed methods design was used. Participants completed a modified food frequency questionnaire (FFQ) assessing major protein food groups since joining NJSC. Semi-structured interviews explored perceived dietary changes, food literacy, and social engagement. Associations between duration of participation and protein food intake were examined using multivariable-adjusted linear regression models. Interview field notes and transcripts were coded in NVivo 12 and thematically analyzed. (3) Results: Among 41 participants, 36 (88%) were female and 17 (41%) were of White ethnicity. A total of 38 (93%) participants reported that NJSC had a positive impact on their lives, and 27 (66%) reported positive changes in eating habits. In multivariable-adjusted analyses, longer participation in NJSC was associated with higher nut consumption (β = 0.49 servings/day per year; 95% CI, 0.02–0.96). Interviews were completed by 40 participants. Seven themes described program support across psychosocial domains (social network; social support; emotional support and mental health; impact on health) and practical nutritional domains (improved food literacy and skills; food decisions; inclusion of plant-based foods). Participants described applying program knowledge at home and perceived improvements in well-being and cancer-related symptoms. (4) Conclusions: NJSC was perceived by members as beneficial across psychosocial and nutritional domains and supported food literacy and plant-forward dietary choices. These findings contribute to our understanding of how culinary cancer care programs can complement existing cancer support services and provide insights for designing future programs for cancer survivors and their support networks.

## 1. Introduction

Cancer is the second leading cause of death globally, accounting for nearly 1 in 6 deaths in 2020 [1]. Consequently, there is a critical demand to meet the needs of those affected by cancer and the subsequent health repercussions [2,3,4]. Research on cancer and nutrition has primarily focused on disease prevention and management [5,6,7], with a tendency to overlook the importance of food literacy, culinary skills, and shared meals [8]. Culinary interventions have been shown to be an effective method to improve self-efficacy for dietary behavior change [9]. As the field of cancer care expands and identifies the social and emotional benefits of support programs [10,11], there emerges an opportunity for these programs to deliver evidence-based nutrition education.

Cancer and its treatments increase the risk of malnutrition [12], with eating behaviors and nutrition being linked to patients’ physical and emotional health [13,14,15]. Programs that focus on nutrition and the practical application of nutrition through culinary skills can support food literacy, dietary behavior change, and improved health outcomes among those with cancer [16]. These programs may also counter conflicting nutrition information commonly encountered by individuals affected by cancer, as many survivors report confusion about nutrition advice from media and other non-expert sources [17].

Shared meals have also been observed to improve nutrition, while also providing a sense of community that can alleviate feelings of isolation [18], often experienced by cancer patients [19]. The act of eating together can strengthen emotional bonds and help individuals cope with their diagnosis, contributing to a more holistic approach to recovery. For caregivers, multicomponent interventions that include education, emotional support, and respite care have been shown to reduce caregiver burden and improve psychological well-being [20]. Often overlooked, caregivers may be at higher risk of poor diet quality while providing care [21]. Given that they frequently assume primary responsibility for meal preparation [22], they also represent an important target for implementation of nutrition strategies.

Culinary cancer care programs bring these elements together by providing nutrition and culinary education while leveraging shared meals to foster social connection. Despite these potential benefits, few studies have examined how integrating a culinary cancer care program within community settings can support members and enhance cancer care. Addressing this gap is critical to inform future models of culinary cancer care programs.

The Not-Just-Supper Club (NJSC) has been an ongoing culinary cancer care program since 2012 that delivers nutrient-dense, health-promoting, plant-based meals within Gilda’s Club Toronto (GT), a community organization. NJSC offers a unique model to examine how integrating a culinary program within a community organization can support members and enhance care. The objectives of the present study were to examine how the NJSC supports its members and to provide recommendations to inform future models of culinary cancer care programs by addressing the following research questions:

1. How do members practically apply the nutrition knowledge and skills gained through NJSC, particularly with respect to protein food choices?
2. How does the NJSC programming provide social and emotional support to members?

## 2. Materials and Methods

### 2.1. Study Design

The study employed an explanatory sequential mixed methods design conducted from September 2017 to January 2019. Participants were recruited from the GT community. Recruitment was conducted through verbal invitations from the principal investigator (PI) and other GT staff, as well as through recruitment emails and posters displayed at GT. This study was an observational evaluation of an existing community-based culinary cancer support program. The program did not prescribe individualized meal plans, caloric targets, or weight-related goals; rather, it emphasized whole, minimally processed foods with a focus on plant-based protein sources and was delivered within a group-based educational setting.

Eligible participants were adults (≥18 years) who were members of GT, had attended at least two NJSC events, and were either living with cancer, had a household or family member living with cancer, or had recently experienced the death of a household or family member from cancer. The inclusion of caregivers in the study ensured the findings represent the lived experiences of all members.

A study team member (JS) contacted eligible individuals by email or phone to confirm eligibility and obtain informed consent prior to participation. Once enrolled, participants completed questionnaires to collect demographic and dietary data. Subsequently, semi-structured interviews were conducted to assess participants’ self-reported changes in dietary behavior and social engagement resulting from their participation in the NJSC. A descriptive phenomenology was selected as the qualitative model to understand the personal experiences of NJSC members [23,24]. Figure S1 presents participant flow. One participant completed the baseline and FFQ components but did not attend the interview and was lost to follow-up.

### 2.2. Program Description

The NJSC is a culinary cancer care program which delivers nutrient-dense, health-promoting, plant-based meals for GT members in a socially supportive environment. GT is a not-for-profit cancer organization [25] that provides free social, emotional, and nutritional support to those who have been touched by cancer, including culinary nutrition programming, and the NJSC program is one example. Since 2012, the NJSC program has been managed by Chef Amy Symington, her team of community volunteers and her students from the Culinary Management Nutrition Program at George Brown College in Toronto, Canada. NJSC’s main objective was to nourish, inspire, and educate members on the benefits of eating and cooking for cancer prevention and management.

Originally beginning in 2012 until the start of the COVID-19 pandemic in March 2020, approximately 50–75 NJSC members and their loved ones would meet weekly to share health-promoting meals together. The program welcomed those touched by cancer (i.e., cancer survivors, caretakers, and/or their family members) to participate in a food demonstration, learn about cancer nutrition, and dine with others in a social setting. NJSC provided recipes (Figure S2) in addition to nutrition information on a variety of topics including foods to consume for cancer prevention and foods to consume for the management of various side effects and symptoms of cancer and its treatment (Figure S3).

### 2.3. Plant-Based Meal Program

The meal program provided to NJSC patrons, which consisted of a soup, salad, main, pasta and dessert, focused on the most recent cancer nutrition guidelines and took into consideration attendees’ dietary needs, restrictions and allergies (Figure S4). Menus were informed by current cancer nutrition evidence [26,27,28,29,30,31] and guidelines [32,33], emphasizing whole plant-based ingredients, including fruits, vegetables (particularly leafy greens and cruciferous, red and orange vegetables), whole grains and high-quality plant-based proteins, and minimizing sugar-sweetened beverages, processed meats, and red meats. Plant protein foods such as nuts, seeds, pulses (beans, chickpeas, lentils, etc.) and soy foods (tempeh, tofu, edamame, etc.) were a major focus of the program, as these foods have been associated with health benefits related to cancer [31,34,35,36] and other chronic diseases [37,38,39] and are not commonly consumed by most Canadians [40]. These plant protein foods including nuts, seeds, pulses, and soy foods were regularly incorporated into program recipes and were featured in the majority of shared meals and cooking demonstrations. Members were shown plant-based adaptations of familiar dishes, demonstrating how foods to limit, such as red meat, can be substituted with options like tempeh or lentils [41].

### 2.4. Dietary Intake of Protein Foods

Dietary intake of major protein food groups since joining the NJSC program was assessed using a 53-item modified food frequency questionnaire (FFQ) based on the European Prospective Investigation of Cancer (EPIC) FFQ [42]. Food items were grouped by protein source including animal-based foods—(1) red meat, (2) fish, (3) dairy, and (4) eggs—and plant-based foods: (5) nuts, (6) seeds, (7) pulses, and (8) soy foods. Consumers were defined as participants reporting intake of the specified food group at least once per month.

### 2.5. Semi-Structured Interviews

Interviews were one-on-one, were approximately 15 min in duration, and followed a semi-structured interview guide (Table 1). All interviews were conducted by the same trained interviewer (JS), minimizing potential variations. JS had experience conducting qualitative research interviews but no previous relationship with NJSC participants. All interviews were conducted in English. Field notes were taken at the time of the interview (JS). Most interviews were audio-recorded to facilitate cross-referencing with field notes; however, recording was optional. Field notes were collected for all interviews.

### 2.6. Quantitative Data Analysis

Continuous normally distributed variables were expressed as mean and standard deviation (SD) and categorical variables expressed as number of cases and percentage. Ethnicity was self-reported and collected by an open-ended questionnaire item and grouped following contemporary race/ethnicity reporting guidelines [43]. Food servings were expressed as servings/day with values presented as mean (SD). To evaluate the linear correlations between food groups and time spent at NJSC, Pearson correlation coefficients were used.

Crude and multivariable-adjusted linear regression analyses were used to examine the associations between reported food group intake (servings/day) and duration of participation at NJSC (years) while accounting for confounders. Covariates included age (continuous), sex (female; male), ethnicity (Asian; Black; Other; White), and body mass index (kg/m^2^).

Exploratory subgroup and sensitivity analyses were conducted. Sex-stratified analyses were performed to evaluate whether the association between time spent at NJSC (years) and nut intake (servings/day) differed between females and males, with effect modification assessed using a multiplicative interaction term in multivariable-adjusted linear regression models. Differences in mean soy intake between participants with breast cancer and those without breast cancer were assessed using two-sample *t*-tests. Nut subtype analyses were also conducted using Pearson correlation coefficients and multivariable-adjusted linear regression models. All statistical analyses were conducted using R (version 4.4.2; R Foundation for Statistical Computing, Vienna, Austria) and were two-sided, and *p* < 0.05 was considered statistically significant.

### 2.7. Qualitative Data Analysis

The qualitative data aimed to elucidate the quantitative results and provide insights into participants’ lived experiences with NJSC [44]. The field notes and transcripts were reviewed independently by two researchers (JS and BJH) to verify the accuracy of participant expressions and responses. Commencing with coding on a line-by-line basis, statements were systematically coded and organized. Discrepancies were resolved by consensus or arbitration by the senior author. Upon reaching consensus on the coding, BJH and JS collaboratively examined the coded transcripts to identify and develop overarching themes using a six-step approach [45,46]. NVivo version 12 Plus [47] was used to facilitate data management and analysis. After this, discussions among three researchers (AS, JS, and BJH) led to amendments and refinements in the codes. These themes underwent further review and refinement and were accompanied by written descriptions.

To enhance credibility, peer debriefing sessions were conducted during the analysis process, involving JS and BJH. This iterative discussion aimed to support a thorough and inclusive interpretation of the data collected, ensuring that diverse perspectives were considered.

Quantitative and qualitative data were analyzed separately. The results of both methods were then compared to determine the degree to which they converged and diverged [48].

### 2.8. Ethical Approval

The project underwent a full ethics review board and research ethics approval was obtained from George Brown College’s research ethics committee (#6004415). All study procedures were in accordance with the ethical standards of George Brown College and the Social Sciences and Humanities Research Council.

## 3. Results

### 3.1. Participant Characteristics

Table 2 presents the baseline characteristics of included participants. Forty-one participants completed the questionnaire and 40 completed the interview. Most participants were female (n = 36; 88%), were 57 ± 16 years of age (mean ± SD), self-identified as White (n = 17; 41%), had a BMI of 26.3 ± 7.5 kg/m^2^, and had spent 1.8 ± 2 years at NJSC. The most common cancer history diagnosis was breast (n = 10; 24%), followed by ovarian (n = 4; 10%). Some participants did not specify their cancer diagnosis (n = 5; 12%), while others self-identified as caregivers and/or members of the cancer bereavement group (n = 13; 32%).

### 3.2. NJSC and Protein Foods

Table 3 summarizes reported consumption, mean (SD) intake, and correlations between food group intake (servings/day) and duration at NJSC (years). Both animal- and plant-based protein food groups were widely consumed by participants (>80%). Time spent at NJSC was positively correlated with nut intake (r = 0.45, *p* = 0.003), whereas no other protein food group demonstrated a significant correlation with duration of participation.

In the crude linear regression analyses, each year of participation in NJSC was associated with a 0.55 servings/day higher intake of nuts (β = 0.55 servings/day per year; 95% CI: 0.20, 0.91). For all other food groups, adjusted regression estimates included the null and results remained consistent after adjustment for confounders (Table S1).

**Figure 1:**
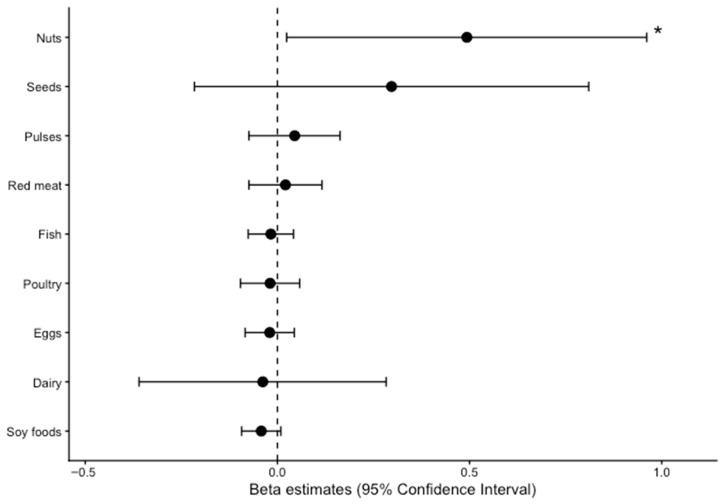
Associations of food groups and duration at Not-Just-Supper Club. Solid lines are values of β coefficients and 95% CI estimated using linear regression models for each year of participation at NJSC and servings/day of protein foods among participants with complete covariate data (n = 37). The models were adjusted for age, sex, ethnicity, and body mass index. Crude and adjusted values can be found in Table S1. * Statistically significant (95% CI does not include the null).

Figure 1 presents multivariable-adjusted regression analyses between duration (years) of participation at NJSC and food group intake among participants with complete covariate data. Models were adjusted for age, sex, ethnicity, and body mass index. Each additional year of participation at NJSC was associated with a higher intake of nuts, corresponding to approximately one-half serving per day (β = 0.49 servings/day per year; 95% CI: 0.02, 0.96). For all other protein food groups, adjusted regression estimates included the null.

### 3.3. Subgroup and Sensitivity Analysis

Table S2 presents the exploratory analyses examining whether associations between time at NJSC and nut intake differed by sex. There was no statistical evidence that the association between time at NJSC and nut intake differs by sex (*p* for interaction = 0.42). Table S3 presents soy intake by cancer subgroups. Soy intake was not statistically different among participants with breast cancer compared with those without breast cancer (0.33 vs. 0.17 servings/day; *p* = 0.30).

Table S4 presents consumption, mean intake, and Pearson correlations between time spent at NJSC and intake of nut types (servings/day). Consumption varied by nut type, with almonds and walnuts being the most consumed and having the highest in mean intake, while intake of hazelnuts and Brazil nuts were reported by fewer participants. Time at NJSC was positively correlated with intake of hazelnuts, walnuts, and pecans (hazelnuts: r = 0.39, *p* = 0.012; walnuts: r = 0.45, *p* = 0.004; pecans: r = 0.35, *p* = 0.029). No significant correlations were observed for peanuts, almonds, or Brazil nuts (all *p* > 0.05).

**Figure 2:**
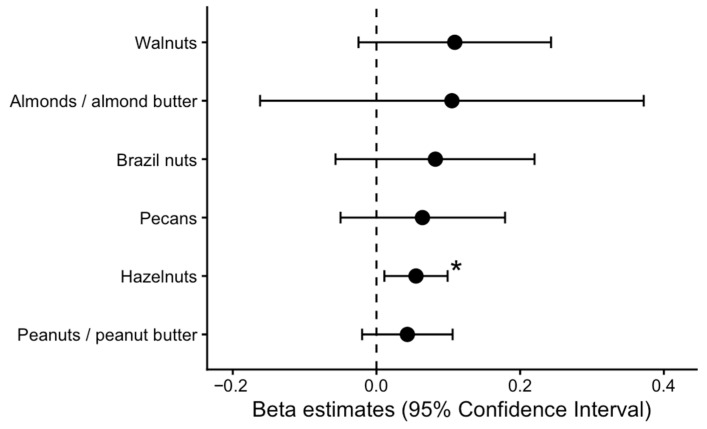
Associations of nut types and duration at Not-Just-Supper Club. Solid lines are values of β coefficients and 95% CI estimated using linear regression models for each year of participation at NJSC and servings/day of nut types among participants with complete covariate data (n = 37). The models are adjusted for age, sex, ethnicity, and body mass index. Crude and adjusted values can be found in Table S2. * Statistically significant (95% CI does not include the null).

Figure 2 shows multivariable-adjusted regression analyses for time at NJSC and intake of nut subtypes among participants with complete covariate data. Each additional year of participation at NJSC was associated with higher hazelnut intake (β = 0.05 servings/day per year; 95% CI: 0.00, 0.11). Adjusted estimates for walnuts, peanuts, almonds, Brazil nuts, and pecans included the null, although all trended in the same direction. Table S5 presents crude and adjusted associations between participation at NJSC and nut types.

### 3.4. Emergent Themes

**Figure 3:**
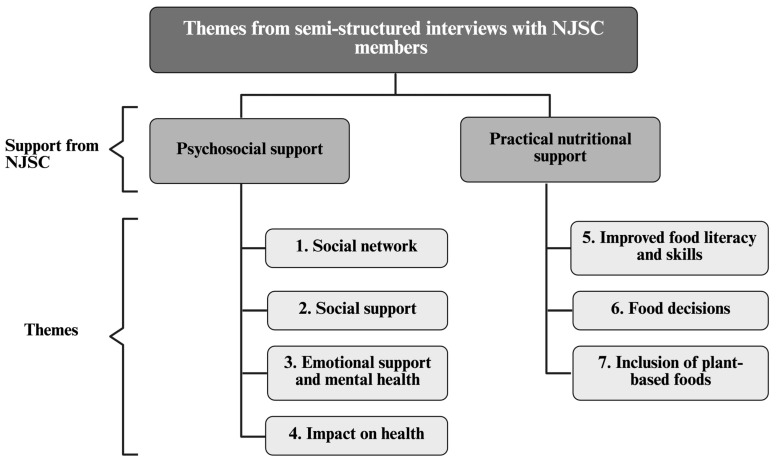
Seven emergent themes from semi-structured interviews (n = 40). Abbreviations: NJSC, Not-Just-Supper Club.

Figure 3 presents the seven themes that were identified describing how NJSC supports its members. Four themes captured psychosocial forms of support: social network; social support; emotional support and mental health; and impact on health. Three themes described the practical nutritional support provided by the program: improved food literacy and skills; food decisions; and inclusion of plant-based foods. Table 4 presents representative quotes for each theme.

Theme 1: Social Network

GT provides an opportunity for members to meet through NJSC, where group interactions create positive experiences and increase social connections. A social network can be defined as a structure composed of a set of actors, some of whose members are connected by a set of one or more relations. NJSC was described as a “family of friends” (Participant 12), and that it’s food is a “fellowship” (Participant 16) and as a conduit for people to come together and learn about nutrition concurrently. Some members reported that NJSC reminded them of positive family or cultural memories. Participant 11 said, “Well it has a positive impact, like eating together. If you look at my family, culture, or habits, we always eat together… it’s just healthier for the soul and the body.” Conversations between members included strategies to manage cancer-related symptoms and non-cancer topics. Members felt comfortable and welcomed because they could talk to others in a social setting. Certain participants extended their acquired knowledge beyond the NJSC by either replicating the dishes they enjoyed or engaging in discussions about recipes, nutrition, and food-related advice.

Theme 2: Social Support

NJSC provided members with social support for different individuals, including family, friends, and staff. Social support can be defined as social interactions or relationships that provide individuals with actual assistance or with a feeling of attachment to a person or a group that is perceived as caring or loving. For those who often eat alone, NJSC may be considered a part of their treatment because it “is the only time [they] eat meals with others. The social interaction helps [them] get out of the house” (Participant 38) and reduces social isolation that can be brought on by a cancer diagnosis. Members reported sharing the nutrition information and recipes they learned with family, friends, or a primary caregiver. One member said, “After my mom’s treatment appointments, I would come here to have supper…and I would get ideas of what foods to prepare for my mom” (Participant 39). Some members have limited social support and reported challenges related to maintaining an intake of nutritious foods. One member shared, “My mom passed away in April. Now I have had significant weight loss… before my mom used to take care of me where I would have three regular meals a day” including salads and soup (Participant 10).

NJSC volunteers and staff are an important social support to members. The volunteers and staff are not only “amazing, polite and helpful” (Participant 31) but also knowledgeable and are considered a useful resource. In addition, Chef Amy was considered as an important individual who provided social support, shared nutrition knowledge, and taught culinary skills. According to one participant, “Supper Club, from Chef Amy’s time, made life interesting. Prior to that, would never touch anything… I am hyperglycemic. She taught me how to appreciate dessert… I learned a lot from my favorite chef!” (Participant 1).

Theme 3: Emotional Support and Mental Health

Caregivers and individuals who live with cancer reported that NJSC provided an emotionally supportive space that was beneficial to their mental health and well-being. One member described this positive impact and said, “Right now, the Supper Club makes me more focused to eat properly” (Participant 2). Similarly, Participant 20 said, the NJSC programming was a “Nice way to ease into the experience, into coming here [GT]. It’s emotionally intense coming to Gilda’s. It’s comforting and relaxing to start with the Supper Club.” More specifically, some participants noted that participating in NJSC is “a stress relief tool. It’s meditation, it brings you away from what you’re experiencing” (Participant 28).

Theme 4: Impact on Health

A total of 38 (93%) of participants reported that NJSC has had a positive impact on their lives. A total of 15 (37%) had a self-reported reduction in cancer-related symptoms. From a more holistic perspective, 11 (27%) participants said that joining NJSC made them “feel well-nourished” (Participant 32). One participant said, “The smell of the food is very nurturing. It makes me feel good and comforted” (Participant 34).

Common cancer-related symptoms such as low energy and poor digestive health were experienced by several members. Some participants reported feeling more energized because of eating healthier at NJSC and noticed improvements in their digestive health. Participant 36 said, “Being able to go and have a home-cooked, healthy meal was really nice, both because it was good food that gives me energy… and it’s a great stress reducer.”

Theme 5: Improved Food Literacy and Skills

Participants identified that NJSC increased their awareness about nutrients in foods and portion sizes, which resulted in more mindful eating. A total of 12 (29%) reported changes in their behavior. The education also enhanced or reinforced what participants already knew about nutrition. A shared sentiment among participants was captured by one participant who said, “it helps motivate to keep me making the changes I’ve been making. It encourages me to keep on the path that I have chosen for myself, which is mostly vegetarian” (Participant 16).

Most individuals who reported no change in their food choices were already eating foods similar to those prepared at NJSC. Participant 7 shared an illustrative perspective and said, “I eat very similar to what they already serve, I eat truckloads of vegetables”.

Participants reported being more interested and motivated to learn about nutrition and health-promoting practices after attending NJSC. A common sentiment was captured by one participant who said, “Knowing about these foods first, and their nutritional properties, would encourage me to eat more of them with a fabulous recipe that can be provided” (Participant 28).

From increasing food literacy (e.g., reading food labels and recipes) to developing food skills (e.g., sprouting beans and using small appliances), participants said they started to learn new food skills and techniques through participating in NJSC. For example, the information shared through NJSC taught participants how to read food labels and recipes. Participant 1 said, “I learned to check what’s in the recipes, and instead of saying “no” I don’t eat that, …, I ask what is in it.” Many participants became more adventurous with their own cooking and conscious about what ingredients they use. Some participants learned new ways to combine ingredients and found it “very exciting to see the flavors and combinations [at NJSC]” (Participants 29 and 30). Other participants learned to use alternative cooking techniques to increase the nutrients in meals, such as Participant 11: “I’m more conscious and cautious with what I use and how I use it. Typically, we cook fresh anyway, but it’s less frying, now more roasting in the oven”.

Not all participants knew how to cook, which was often cited as a barrier to implementing change. Some individuals were still interested in learning more despite their limited cooking abilities. In addition, many participants expressed interest in joining cooking classes to improve food literacy and skills that would enable them to live healthier. Participants expressed that they “feel like [they] are doing something positive for [their] situation” (Participant 18) and are more confident in their ability to prepare nutritious foods to support their health after attending the NJSC. For example, Participant 5 said, “I want to learn what to eat from Chef Amy and learn to prepare for myself. Chef Amy won’t cook for me forever.”

Theme 6: Food Decisions

Participants were able to make healthier food decisions based on what they learned in NJSC, particularly increasing consumptions of culturally diverse and anti-cancer foods. A total of 27 (66%) of participants reported that NJSC has positively impacted their eating habits. Participant 12 said, “I do pay more attention to food labelling, so it definitely has made me more of a judgmental shopper…and makes me have more confidence and diversity in my food selection.”

Furthermore, by eating more nutritious foods, participants reported various changes in their behavior and health. One participant said, “I was taking a lot of other supplements, and it was costing me a fortune… however, I think it’s really good to be promoting nutrition and plant-based diets, because we can get more benefits through nutrition than supplements” (Participant 18). Participant 25 illustrated how their vegetable consumption increased and said, “I never ate vegetables before Supper Club, so yes, [my diet has] improved, when I do eat the vegetables now, I go to the bathroom more.”

Theme 7: Inclusion of Plant-based Foods

As recommended by cancer nutrition guidelines, many participants shared that they were more interested in eating plant-based foods since joining NJSC. Some participants reported wanting to learn more about consuming plant-based proteins to ensure adequate dietary consumption. Participant 10 said, “Especially learning about alternative protein sources… I’d like to learn more to make sure that I am getting enough protein, and that I am getting it from non-animal sources.”

Individuals also reported adding more vegetables and plant-based recipes to their regular diet. For example, one participant said, “Even if I do prepare chicken, I usually wouldn’t have salads, but now I am trying to add more vegetables to my plate or my son’s plate. That is due to me coming to Supper Club. I am learning all of that here” (Participant 25).

## 4. Discussion

### 4.1. Principal Findings

In this mixed methods study of 41 members of the NJSC, we examined how integrating culinary cancer care programming within a community-based organization can support members. Most participants reported that the program positively impacted their lives and eating habits, and longer participation in the program was associated with higher nut intake, with each additional year of participation being associated with an additional half serving per day of nuts in multivariable-adjusted models, after adjusting for covariates. However, there was no strong evidence of associations for the other protein food groups.

The qualitative findings complemented these results by providing comprehensive insights into participants’ experiences, their motivations for participation, and potential behavior changes including the application of newly acquired nutrition and culinary knowledge. Seven themes described how NJSC supports members through psychosocial domains (social network, social support, emotional support and mental health, and impact on health) and through practical nutritional support (improved food literacy and skills, food decisions, and inclusion of plant-based foods). Participants described applying program knowledge at home and perceived improvements in well-being and cancer-related symptoms. Together, these convergent findings suggest that a culinary cancer program can enhance the support members receive from community organizations through both psychosocial pathways and practical nutrition-related mechanisms.

Plant protein foods including nuts, seeds, pulses (beans, chickpeas, lentils, etc.) and soy foods (tempeh, tofu, edamame, etc.) were all a major focus of the NJSC program. The reason nuts were positively associated with duration at NJSC but no associations were observed for other plant protein foods needs to be further explored. Higher adherence to nuts compared to other plant foods has been observed by others [49], possibly due to nuts requiring less preparation and being highly palatable. Nuts were included in approximately one third of the dishes prepared for members and were emphasized in nutrition education sessions. These findings are encouraging as nut consumption has been inversely associated with cancer risk [34]. When considering nut subtypes, participation at NJSC was positively correlated with intake of several nut subtypes, including hazelnuts, walnuts, and pecans, yet in multivariable-adjusted analyses, only hazelnut intake remained positively associated with duration of participation. Seeds also trended in the same direction as nuts, but there was no strong evidence of associations with estimates including the null.

When considering other protein groups, soy intake trended inversely with duration at NJSC. A subgroup analysis explored whether soy consumption differed among those who reported breast cancer compared to the rest of the population and found no evidence of lower soy intake among participants with breast cancer. These findings are reassuring given misconceptions surrounding soy having estrogenic effects and the lack of evidence supporting concerns about breast cancer risk [50,51]. Both analyses of nut subtypes and soy intake by cancer subgroups were exploratory and should be interpreted cautiously.

Participants also reported increasing their intake of fruits and vegetables after attending NJSC. These changes could not be quantitatively evaluated because the abbreviated protein-focused FFQ did not assess fruit and vegetable consumption. Therefore, we are unable to determine whether perceived increases translated into measurable dietary change. However, this perceived dietary change remains notable, as higher fruit and vegetable intake is strongly recommended by cancer nutrition guidelines [1]. Future evaluations should incorporate a more comprehensive dietary assessment to quantify changes in all measures of dietary quality

### 4.2. Comparison with Previous Work

This study is one of the few to examine the experiences of participants in an ongoing culinary cancer care program in Canada. A recent scoping review by Johnston et al. identified 37 group-based nutrition education and cooking programs for people affected by cancer, highlighting growing but still limited evidence in this area [52]. The review noted substantial heterogeneity in study design, especially delivery format (in-person vs. virtual) and outcome measures. Most interventions (n = 21) were conducted in the US, with only four studies in Canada, were short-term (typically 6–12 weeks), and focused primarily on behavioral or psychosocial endpoints rather than dietary patterns.

Several interventions have employed structured, time-limited programs delivered virtually or in-person with quantitative pre–post assessments. Huang et al. conducted a 9-week virtual teaching kitchen intervention focused on plant-based and Mediterranean dietary patterns among 102 breast cancer survivors and reported improvements in mindful eating behaviors in a pre–post design [53]. Likewise, Allen-Winters et al. piloted an 8-week culinary medicine program in a small cohort of children/caregiver dyads (n = 9 dyads) demonstrating improvements in dietary choices and cooking confidence in a pre–post design [13]. Barak-Nahum et al. evaluated a 10-week culinary group intervention within a controlled trial among 109 adult cancer patients and found improvements in dietary behaviors, health-related quality of life, and psychological well-being when compared with a control group [54].

More recent work not included in the Johnston review includes Pritlove et al., who conducted a pilot culinary nutrition intervention among gynecologic cancer survivors (n = 53) in Canada treated with pelvic radiotherapy, reporting improvements in digestive symptoms and self-reported dietary practices in a pre–post design [55].

Qualitative work has provided insight into survivors’ lived experiences with food following cancer treatment. O’Callaghan et al. conducted an interview-based qualitative study with 20 cancer survivors and reported that post-treatment nutrition experiences were shaped by evolving beliefs about food, uncertainty, and the desire for practical, trustworthy guidance [56]. Similarly, Murat-Ringot et al. described an observational pilot of peer-led plant-based cooking workshops among six post-cancer participants, highlighting perceived improvements in dietary practices, social connectedness, and empowerment [57]. These qualitative studies emphasize the psychosocial and experiential dimensions of nutrition after cancer, complementing the behavioral outcomes reported in structured interventions.

In contrast to these interventions, NJSC represents an ongoing, real-world community-based culinary cancer care program embedded within a support organization. By including both cancer survivors and their support networks in the same program, our findings represent the lived experiences of all members in the NJSC. While less common, the inclusion of caregivers in culinary cancer care research offers a more holistic approach to support, acknowledging the interconnectedness of the patient’s well-being and the well-being of their support network. In a scoping review of group nutrition education and cooking programs for those affected by cancer, only four of the 37 programs invited caregivers to attend [52]. This limited inclusion represents a notable gap as caregivers may be at a higher risk of poor dietary intake while providing care [21] and often assume primary responsibility for meal preparation [22]. By involving caregivers, programs can enhance the application of the intervention and may encourage sustained long-term healthy eating habits within the household.

### 4.3. Strengths and Limitations

This study has several strengths, including its mixed methods design, which enabled integration of quantitative dietary data with qualitative insights from interviews to provide a more comprehensive understanding of participants’ experiences. It also included both cancer survivors and members of their support networks, broadening the range of perspectives captured in prior studies. Notably, the study was conducted within an established culinary cancer care program that had been operating for more than five years before study initiation, ensuring that the findings reflect a feasible, real-world model.

Several limitations should also be acknowledged. The small sample size limits statistical power, particularly for multivariable regression and subgroup analyses, and warrants cautious interpretation of quantitative findings. Restriction of our participant population to current NJSC members may have resulted in a predominance of female participants (88%), limiting the generalizability of our findings to male cancer patients and caregivers and to individuals who do not enroll in or remain engaged with culinary cancer care programs. The lack of randomization and intention-to-treat framework also introduces potential selection bias.

Additionally, this study was cross-sectional and dietary data were collected at a single time point, which precluded direct assessment of sustained changes over time. Larger, adequately powered longitudinal studies with repeated dietary assessments and clinical measures are needed to evaluate the durability of behavioral changes and potential long-term health impacts of culinary cancer care programs. The FFQ also included a limited number of food items, restricting dietary assessment primarily to protein foods. These design choices were made to minimize participant burden during a particularly challenging period [58]. Moreover, reliance on an FFQ to capture dietary intake introduces the potential for recall and reporting bias [59], which may affect reliability.

Additionally, this study did not formally measure individual at-home use of the evidence-based resources provided during NJSC programming. Future evaluations should incorporate a structured assessment of resource utilization to better understand its impact on dietary behavior change, cancer nutrition knowledge, and food literacy. Furthermore, the study may have benefited from participants’ insights into the accuracy of the transcriptions and their interpretations of the data, which could have enhanced credibility to findings [60]. While debate exists regarding the role of participant transcript review, it is widely acknowledged as one strategy for supporting rigor in qualitative inquiry.

### 4.4. Implications for Practice and Recommendations for Future Programs

These findings can inform the development of future cancer care programs, ensuring they are designed to motivate and support positive behavior changes related to health and nutrition. The results suggest that culinary cancer care programs can complement existing support services by pairing evidence-based nutrition and nutrient-dense meals with regular opportunities for social connection.

To support translation of the program beyond the NJSC setting, two freely available resources are now hosted on GT’s website [25], including a cancer nutrition guide [61] and a companion cookbook [62], which were developed as part of the project along with many other resources. Additionally, a cookbook was developed through the program with recipes tested by NJSC members [63]. These resources may be used by cancer care affiliates to implement similar culinary cancer care programs.

Drawing from insights gained through NJSC, five recommendations for future cancer programming are proposed.

(1) Integration of food in cancer programming

Culinary cancer care programs should be incorporated into broader cancer programming. Integrating regular nutrition and culinary components can facilitate the development of food literacy and skills among cancer survivors and their families/caretakers. Eating meals together also helps to build a sense of community and belonging among participants, enhancing the multidimensional support of cancer programs.

(2) Nutrition resources in cancer programming

Community-based culinary programs that bring members together around food may provide an effective setting for delivering evidence-based nutrition. In this context, members may be more receptive to nutrition guidance delivered through trusted, shared experiences rather than information accessed through impersonal sources. Given the prevalence of misinformation surrounding cancer and nutrition [17], providing evidence-based supplementary resources is particularly important. Cancer programs should therefore provide accessible, evidence-based nutrition resources that reinforce program objectives to encourage the consumption of health-promoting foods at home and to support recently gained culinary skills.

(3) Tailoring the program to members and support networks

Culinary cancer care programs should be tailored to the specific needs of an organization’s members and their support networks. This may include offering virtual cooking and nutrition sessions for immunocompromised participants or providing targeted nutrition education with practical handouts that address the dietary needs and challenges of the population served. Where appropriate, programs should consider engaging caregivers and family members, who often play a central role in meal preparation and food-related decision-making. Involving support networks may improve the feasibility of dietary changes, foster shared understanding of nutrition-related challenges, and promote supportive home food environments. Collectively, these approaches may enhance program relevance, reduce isolation, and support sustained dietary behavior change.

(4) Interdisciplinary collaboration in cancer programming

Culinary cancer care programs should be developed and delivered through collaboration among professionals with expertise in culinary and nutritional science, including chefs, oncologists, and dietitians. Integrating expertise across disciplines may enhance the accuracy and acceptability of nutrition messaging while ensuring programs address both the physical and psychosocial needs of individuals affected by cancer.

(5) Importance of continued research on culinary cancer programs

Further research is needed to evaluate the effectiveness, scalability, and long-term impact of culinary cancer care programs on members’ dietary behaviors, health, and well-being. Longitudinal multifactorial designs can assess how integrating psychological and behavioral components may enhance program effectiveness. Future studies should also explore virtual or hybrid delivery models for culinary cancer care programs that maintain opportunities for social connection while the sharing of knowledge and skills.

## 5. Conclusions

Participation in a culinary cancer care program was associated with perceived improvements in dietary behaviors and cancer-related symptoms, alongside overwhelmingly positive participant experiences. Longer participation at NJSC was also associated with higher nut intake. The identified themes from interviews highlighted how NJSC supported members’ health and well-being through psychosocial and nutritional pathways, including the practical application of new nutrition knowledge.

These findings underscore the potential of culinary cancer care programs to promote holistic well-being by addressing nutritional, emotional, and social dimensions of cancer care. The development of evidence-based resources and recommendations as part of the project provides tangible tools that can be utilized by cancer care organizations implementing similar culinary programs. Overall, these findings extend the existing literature, contributing a more comprehensive understanding of participant experiences and informing the design of future programs.

## Figures and Tables

**Table 1:** Primary semi-structured interview questions.

	Question
1	Have you developed any new eating habits or eating routines since becoming a Not-Just-Supper Club member? If so, please describe.
2	Do you find that you have changed the way you prepare and eat foods at home since joining the Not-Just-Supper Club? If so, please describe.
3	Do you find that you share food ideas and recipes that you’ve tried at the Not-Just-Supper Club with family and friends? If so, please describe.
4	Do you feel your cancer-related symptoms or treatment side effects have improved since you’ve been attending and eating at Not-Just-Supper Club? If so, please describe.
5	Do you feel more interested and motivated to learn about nutrition tips and health-promoting practices after attending the Not-Just-Supper Club? If so, please describe.

**Table 2:** Characteristics of NJSC participants.

Characteristic	n = 41
Age (years) ^a^, mean (SD)	57.4 (15.8)
Sex	
Female	36 (88)
Male	5 (12)
Ethnicity ^b^	
White	17 (41)
Other	11 (27)
Asian	6 (15)
Black	5 (12)
Did not answer	2 (5)
Current smoker	
Yes	3 (7)
No	38 (93)
BMI (kg/m^2^), mean (SD)	26.3 (7.5)
Duration at NJSC (years), mean (SD)	1.8 (2.0)
Cancer diagnoses	
Breast	10 (24)
Ovarian	4 (10)
Bladder	1 (2)
Esophageal	1 (2)
Lymphoma	1 (2)
Lung	1 (2)
Renal	1 (2)
Salivary	1 (2)
Choriocarcinoma	1 (2)
Uterine	1 (2)
Multiple	1 (2)
Did not answer	5 (12)
None ^c^	13 (32)

Values expressed as No. (%) unless otherwise indicated. Abbreviations: BMI, body mass index; GT, Gilda’s Club Greater Toronto; SD, standard deviation. Questions with missing entries were classified as “Did not answer”. ^a^ Presented mean is for n = 39 as two participants chose not to disclose year of birth. ^b^ Ethnicity was self-reported and collected via an open-ended questionnaire item. For analysis, responses indicating Caucasian, European Caucasian, Canadian Caucasian, British Caucasian, Italian, Scottish, WASP, and Ashkenazi were categorized as “White”. Responses indicating Chinese, Japanese, or Southeast Asian were categorized as Asian, and responses indicating Jamaican, Caribbean, African Canadian, Canadian Black, or Canadian–West Indies were categorized as “Black”. All remaining responses (e.g., Canadian, Spiritually Jewish, and Ecuadorian) and responses indicating more than one ethnicity were grouped as “Other” due to small numbers. Categories were grouped to allow for analysis. ^c^ The program welcomed all members of GC, including family, caregivers, and/or members of the cancer bereavement group.

**Table 3:** Servings of animal- and plant-based protein foods and correlations with time at Not-Just-Supper Club (NJSC) (n = 41).

Protein Group ᵃ	Consumers, n (%)	Servings/day (SD)	Pearson r with Time at NJSC	*p*-Value
Red meat	35 (85.4)	0.41 (0.44)	0.01	0.94
Poultry	33 (80.5)	0.26 (0.40)	−0.07	0.65
Fish	38 (92.7)	0.34 (0.26)	−0.05	0.77
Dairy	35 (85.4)	1.37 (1.83)	0.00	0.99
Eggs	38 (92.7)	0.39 (0.29)	−0.09	0.59
Nuts	41 (100)	1.67 (2.14)	0.45	0.003
Seeds	41 (100)	1.72 (2.44)	0.19	0.25
Pulses	41 (100)	0.56 (0.55)	0.03	0.83
Soy foods	40 (97.6)	0.21 (0.29)	−0.26	0.11

Consumers were defined as participants reporting intake of the specified food group at least once per month. Servings/day values are presented as mean (SD) servings per day. *p*-value is for Pearson correlation. ^a^ Protein food group definitions: Red meat includes beef, burgers, pork, lamb, bacon, ham, luncheon meats, sausages, and meat pies. Poultry includes chicken and turkey. Fish includes fried fish, fish fingers, white fish, oily fish, shellfish, and fish roe. Dairy includes sour cream, yogurt, dairy desserts, cheese, and cottage cheese. Nuts include peanuts, peanut butter, hazelnuts, walnuts, almonds, almond butter, Brazil nuts, and pecans. Seeds include pumpkin, sesame, hemp, chia, flax, and sunflower seeds. Pulses include beans and chickpeas. Soy foods include soy milk, edamame, tofu, and tempeh.

**Table 4:** Representative quotes from semi-structured interviews for emerging themes.

Major Themes	Representative Quotes
Theme 1: Social network	“The social component of Supper Club is so important. I almost eat all of my meals alone, this is the only time I eat meals with others. I felt like going to Gilda’s Club was a part of my treatment. Social interaction helps me get out of the house.” (Participant 38)
Theme 2: Social support	“My chance to try [the recipes] with friends is during potluck, which I started this week. And I learned to be creative, there is so much to create! Whatever I have in the house, I create.” (Participant 33) “I live alone. A lot of my friends live in other parts of the city, or they have partners or family, so I don’t see them often. It is nice to join a group of people at Gilda’s to chat… It’s nice and it has widened my network and contacts in the city; apart from having a nice tasty meal.” (Participant 40)
Theme 3: Emotional support and mental health	“Good food that gives me the energy and for time, it’s a great stress reducer. Gave me more time with people that I am in support group with. Decreased anxiety and stress, and more energy due to the healthy food.” (Participant 36) “Think it’s an amazing club to have, the Supper Club, whether you are a caregiver or someone with cancer, you need somewhere to unwind and breathe.” (Participant 39) “The food is done with love and care, I can feel that” (Participant 14)
Theme 4: Impact on health	“The smell of the food is very nurturing. It makes me feel good and comforted” (Participant 34). “My breast has cleared, I am not having fibrous breast, so if I stay on this diet, I will be clear. My energy is good. And my bowel movements, have been so good. So I eat well so that I am having healthy and textured bowel movements.” (Participant 27)
Theme 5: Improved food literacy and skills	“I learned how to peel ginger with a spoon.” (Participant 14) “Fairly good to begin with, but more conscious of the not-meat options. I always knew legumes and beans are cheap and reasonable to substitute for meat. Every week since I have come here for half of year… I’ve become more conscious.” (Participant 13)
Theme 6: Food decisions	“I try to check in with how I am eating, because of different health issues that have come up. This has re-instilled how to think about what I am eating.” (Participant 15) “Yes. I try to eat healthier. I added things that I learned about at the Supper Club into my diet. Like flax seeds, couscous. Try to make more conscious choices. Particularly anti-cancer foods.” (Participant 39)
Theme 7: Inclusion of plant-based foods	“Learned that meals don’t have to be meat-based/meat component to be filling. It also showed me the gap in my own repertoire of recipes.” (Participant 22)

## Data Availability

The data presented in this study and the analytic code used may be made available upon request from the corresponding author.

## Supplementary Materials

The following supporting information can be downloaded at: https://www.mdpi.com/article/10.3390/nu18050858/s1, Figure S1: Flow of the study participants; Figure S2: Example of an NJSC-developed recipe for a Curry Chickpea Salad Sandwich; Figure S3: Examples of NJSC-developed cancer nutrition resources; Figure S4: Example of the Not-Just-Supper Club Program’s Menu; Table S1: Crude and adjusted associations between participation at Not-Just-Supper Club and major protein foods (n = 41); Table S2: Association between time at Not-Just-Supper Club and nut intake by sex (n = 41); Table S3: Associations of soy intake and breast cancer among participants at Not-Just-Supper Club (n = 41); Table S4: Servings of individual nut types and their correlations with time spent at the Not-Just-Supper Club (NJSC) (n = 41); Table S5: Crude and adjusted associations between participation at Not-Just-Supper Club and nut types (n = 41).
