# Supplementary material for: Culinary Nutrition Programming for Members of a Community-Based Cancer Program
Source: Nutrients. 2026 Mar 6;18(5):858. doi: 10.3390/nu18050858 (PMC12986692; doi:10.3390/nu18050858)
Supplement: Supplementary file 1 [file nutrients-18-00858-s001.zip › nutrients-4151714-supplementary.pdf]

Supplementary Materials

Culinary Programming for Members of a Community-Based Cancer Program

Billie Jane C. Hermosura, Meaghan E. Kavanagh, Jaime Slavin, David J.A. Jenkins, and Amy Symington

Table of Contents

Figure S1. Flow of the study participants. 2

Figure S2. Example of a NJSC-developed recipe for Curry Chickpea Salad Sandwich. 3

Figure S3. Examples of NJSC-developed cancer nutrition resources. 4

Figure S4. Example of the Not-Just-Supper Club Program's Menu. 5

Table S1. Crude and adjusted associations between participation at Not-Just-Supper Club and major protein foods (n=41). 6

Table S2. Association between time at Not-Just-Supper Club and nut intake by sex (n=41). 6

Table S3. Associations of soy intake and breast cancer among participants at Not-Just-Supper Club (n=41). 7

Table S4. Servings of individual nut types and their correlations with time spent at the Not-Just-Supper Club (NJSC) (n = 41). 7

Table S5. Crude and adjusted associations between participation at Not-Just-Supper Club and nut types (n=41). 7

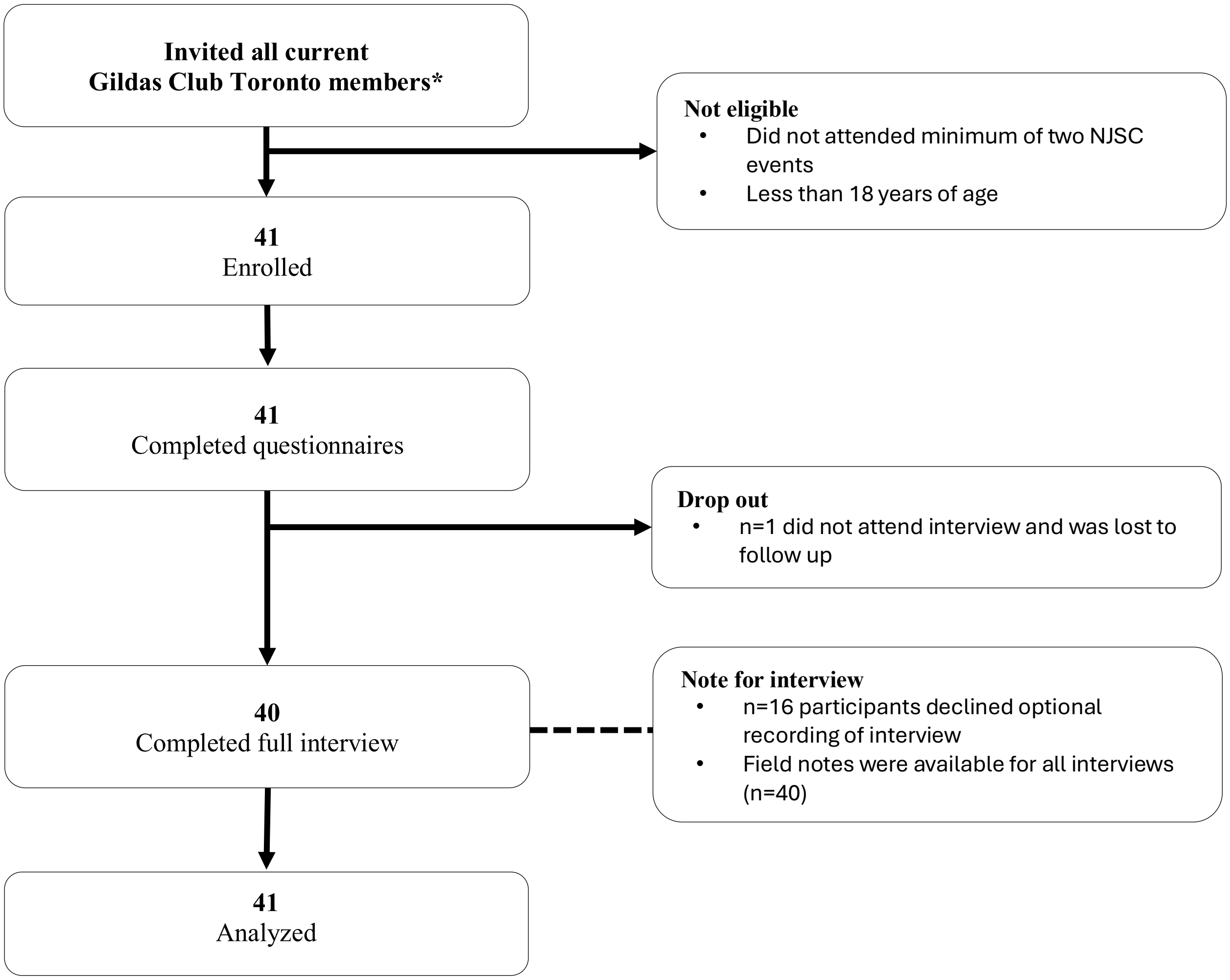

Figure S1. Flow of the study participants.

*Number of eligible GT members or number of those screened was not collected during the study.

Curry Chickpea Salad Sandwich

Emily Goncz & Morgan Campbell
Adapted by Chef Amy Symington

Preparation Time: 15 minutes
Cooking Time: 40 minutes

- Preheat oven to 375°F (190°C).
- Equipment: Food processor, baking sheet, parchment paper

Ingredients

**6 servings**	**24 servings**
1 (50 g) head of garlic	4 (200 g) heads of garlic
2 tbsp (30 ml) grapeseed oil, divided	½ cup (125 ml) grapeseed oil, divided
1½ cups (215 g) sweet potato, cut into 1/2 inch cubes	6 cups (770 g) sweet potato, cut into 1/2 inch cubes
1 tbsp (7 g) curry powder	¼ cup (25 g) curry powder
1½ tsp (4 g) ground coriander	2 tbsp (10 g) ground coriander
1½ tsp (3 g) ground cumin	2 tbsp (10 g) ground cumin
1½ tsp (3 g) paprika	2 tbsp (15 g) paprika
1½ tsp (3 g) turmeric, ground	2 tbsp (10 g) turmeric, ground
3 cups (500 g) unsalted canned chickpeas, drained and rinsed	12 cups (1.74 kg) unsalted canned chickpeas, drained and rinsed
1 stalk (60 g) celery, diced	4 stalks (250 g) celery, diced
¼ cup (60 ml) tahini	1 cup (250 ml) tahini
1½ tbsp (6 g) nutritional yeast	6 tbsp (20 g) nutritional yeast
¾ cup (60 g) thinly sliced green onion	3 cups (160 g) thinly sliced green onion
1½ tsp lemon zest	2 tbsp (7 g) lemon zest
2 tbsp (30 ml) fresh lemon juice	½ cup (125 ml) fresh lemon juice
1/8 bunch (3 g) parsley, finely chopped	½ bunch (12 g) parsley, finely chopped
½ cup (125 ml) vegan mayo	1 cup (250 ml) vegan mayo
1 tsp (5 g) sea salt	4 tsp (20 g) salt
¼ tsp black pepper	1 tsp black pepper
**For sandwiches:**	**For sandwiches:**
12 slices bread, multigrain	48 slices bread, multigrain
1 beefsteak tomato, thinly sliced	4 beefsteak tomatoes, thinly sliced
½ head boston lettuce	2 heads Boston lettuce

Directions

1. Pre-heat oven to 375°F (190°C). Line a baking sheet with parchment paper.
2. Cut the tops of the heads of garlic. Lightly drizzle with 1/4 of the grapeseed oil. Place on prepared baking sheet. Set aside.
3. Toss the sweet potatoes with the spices and the remaining oil. If room permits, spread evenly on the same baking sheet with the garlic. If making 24 servings use an additional baking sheet lined with parchment. Cook for approximately 40 minutes, or until sweet potatoes are fork tender and garlic is golden and soft. Allow to cool slightly before removing garlic skins.
4. Next, in a food processor, add the roasted garlic, chickpeas, tahini, nutritional yeast, lemon zest and juice. Pulse mixture 5 to 8 times (or 15 to 18 times if making 24 servings) until all ingredients are incorporated and chickpeas are slightly broken-down, but still chunky.
5. In a large bowl, mix together roasted sweet potato, chickpea mixture, celery, green onions, parsley, and mayo. Add salt and pepper. Refrigerate for 20 minutes, or overnight.
6. Serve on bread with boston lettuce and sliced tomatoes.

Tips:

1) Longer chilling times will produce better flavour for the filling.
2) Be sure to not over process chickpeas in food processor, as there should still be some semi-whole chickpeas. It should be chunky, and not a puree.
3) For a gluten free option, add filling to zucchini ribbons and roll. Secure with a toothpick.

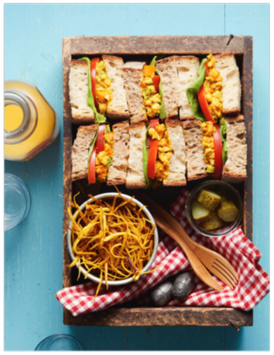

Symbols: kid friendly, good for summer or spring, nut free, high source of fibre, high protein, good for bowel motility

Functional foods present: legumes (chickpeas), orange vegetables (sweet potato), allium vegetables (onions, garlic), spices, turmeric, leafy greens (parsley), seeds (tahini), nutritional yeast, whole grains (whole grain bread), tomatoes

Examples of phytochemicals and anti-oxidants present: carotenoids (sweet potato, leafy greens), allicin (onions, garlic), polyphenols (whole grains), curcumin (turmeric), lycopene (tomatoes)

Intro: The satisfyingly creamy salad sandwiches need not be omitted when eating for chronic disease prevention and management. They can be equal parts indulgent and health promoting, like our Curried Chickpea salad sandwich recipe equipped with homemade mayo and an assortment of crunchy and sweet vegetables. One sandwich contains over 50% of your Recommended Dietary Allowance for fibre and 19 g of protein.

Nutrition Facts
Valeur nutritive
Per 1 sandwich (347 g)
par 1 sandwich (347 g)

**Calories 510**	% Daily Value* / % valeur quotidienne*
**Fat / Lipides** 17 g	23 %
Saturated / saturés 1 g + Trans / trans 0 g	5 %
Omega-3 / oméga-3 0.1 g	
**Carbohydrate / Glucides** 67 g	
Fibre / Fibres 15 g	54 %
Sugars / Sucres 4 g	4 %
**Protein / Protéines** 19 g	
**Cholesterol / Cholestérol** 0 mg	
**Sodium** 490 mg	21 %
Potassium 800 mg	17 %
Calcium 150 mg	12 %
Iron / Fer 6 mg	33 %
Vitamin A / Vitamine A 300 mcg	33 %
Vitamin C / Vitamine C 17 mg	19 %
Vitamin E / Vitamine E 2 mg	13 %

*5% or less is **a little**, 15% or more is **a lot**
*5% ou moins c'est **peu**, 15% ou plus c'est **beaucoup**

Symbols: kid friendly, good for all seasons, high source of fibre, good for bowel motility, gluten free, soy free, nut free,

Functional foods present: parsnips, spices, turmeric, dried herbs

Examples of phytochemicals and anti-oxidants present: carotenoids (dried herbs), curcumin (turmeric)

Figure S2. Example of a NJSC-developed recipe for Curry Chickpea Salad Sandwich.

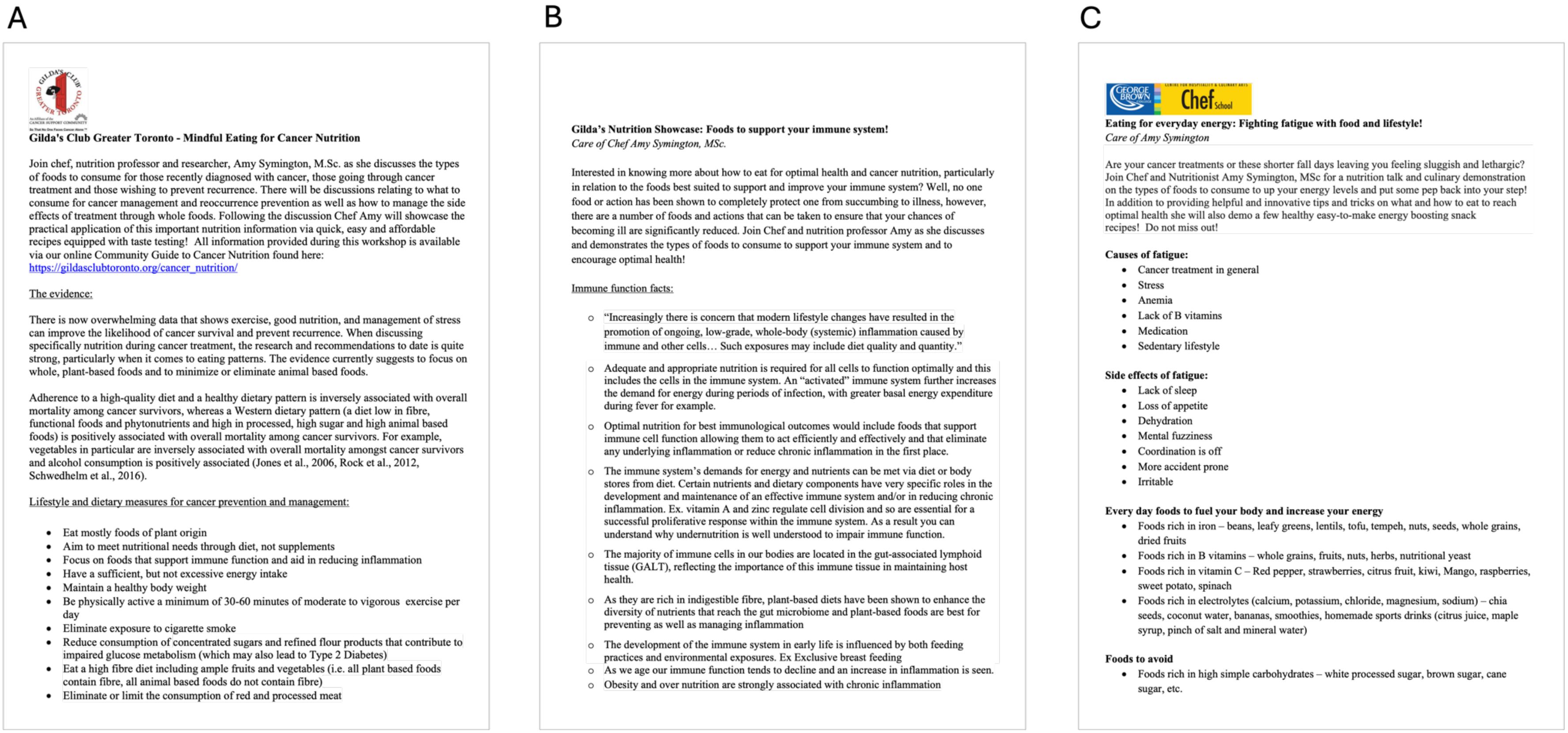

A

Gilda's Club Greater Toronto - Mindful Eating for Cancer Nutrition

Join chef, nutrition professor and researcher, Amy Symington, M.Sc. as she discusses the types of foods to consume for those recently diagnosed with cancer, those going through cancer treatment and those wishing to prevent recurrence. There will be discussions relating to what to consume for cancer management and reoccurrence prevention as well as how to manage the side effects of treatment through whole foods. Following the discussion Chef Amy will showcase the practical application of this important nutrition information via quick, easy and affordable recipes equipped with taste testing! All information provided during this workshop is available via our online Community Guide to Cancer Nutrition found here: https://gildasclubtoronto.org/cancer_nutrition/

The evidence:

There is now overwhelming data that shows exercise, good nutrition, and management of stress can improve the likelihood of cancer survival and prevent recurrence. When discussing specifically nutrition during cancer treatment, the research and recommendations to date is quite strong, particularly when it comes to eating patterns. The evidence currently suggests to focus on whole, plant-based foods and to minimize or eliminate animal based foods.

Adherence to a high-quality diet and a healthy dietary pattern is inversely associated with overall mortality among cancer survivors, whereas a Western dietary pattern (a diet low in fibre, functional foods and phytonutrients and high in processed, high sugar and high animal based foods) is positively associated with overall mortality among cancer survivors. For example, vegetables in particular are inversely associated with overall mortality amongst cancer survivors and alcohol consumption is positively associated (Jones et al., 2006, Rock et al., 2012, Schwedhelm et al., 2016).

Lifestyle and dietary measures for cancer prevention and management:

- Eat mostly foods of plant origin
- Aim to meet nutritional needs through diet, not supplements
- Focus on foods that support immune function and aid in reducing inflammation
- Have a sufficient, but not excessive energy intake
- Maintain a healthy body weight
- Be physically active a minimum of 30-60 minutes of moderate to vigorous exercise per day
- Eliminate exposure to cigarette smoke
- Reduce consumption of concentrated sugars and refined flour products that contribute to impaired glucose metabolism (which may also lead to Type 2 Diabetes)
- Eat a high fibre diet including ample fruits and vegetables (i.e. all plant based foods contain fibre, all animal based foods do not contain fibre)
- Eliminate or limit the consumption of red and processed meat

B

Gilda's Nutrition Showcase: Foods to support your immune system!
Care of Chef Amy Symington, MSc.

Interested in knowing more about how to eat for optimal health and cancer nutrition, particularly in relation to the foods best suited to support and improve your immune system? Well, no one food or action has been shown to completely protect one from succumbing to illness, however, there are a number of foods and actions that can be taken to ensure that your chances of becoming ill are significantly reduced. Join Chef and nutrition professor Amy as she discusses and demonstrates the types of foods to consume to support your immune system and to encourage optimal health!

Immune function facts:

- "Increasingly there is concern that modern lifestyle changes have resulted in the promotion of ongoing, low-grade, whole-body (systemic) inflammation caused by immune and other cells… Such exposures may include diet quality and quantity."
- Adequate and appropriate nutrition is required for all cells to function optimally and this includes the cells in the immune system. An "activated" immune system further increases the demand for energy during periods of infection, with greater basal energy expenditure during fever for example.
- Optimal nutrition for best immunological outcomes would include foods that support immune cell function allowing them to act efficiently and effectively and that eliminate any underlying inflammation or reduce chronic inflammation in the first place.
- The immune system's demands for energy and nutrients can be met via diet or body stores from diet. Certain nutrients and dietary components have very specific roles in the development and maintenance of an effective immune system and/or in reducing chronic inflammation. Ex. vitamin A and zinc regulate cell division and so are essential for a successful proliferative response within the immune system. As a result you can understand why undernutrition is well understood to impair immune function.
- The majority of immune cells in our bodies are located in the gut-associated lymphoid tissue (GALT), reflecting the importance of this immune tissue in maintaining host health.
- As they are rich in indigestible fibre, plant-based diets have been shown to enhance the diversity of nutrients that reach the gut microbiome and plant-based foods are best for preventing as well as managing inflammation
- The development of the immune system in early life is influenced by both feeding practices and environmental exposures. Ex Exclusive breast feeding
- As we age our immune function tends to decline and an increase in inflammation is seen.
- Obesity and over nutrition are strongly associated with chronic inflammation

C

Eating for everyday energy: Fighting fatigue with food and lifestyle!
Care of Amy Symington

Are your cancer treatments or these shorter fall days leaving you feeling sluggish and lethargic? Join Chef and Nutritionist Amy Symington, MSc for a nutrition talk and culinary demonstration on the types of foods to consume to up your energy levels and put some pep back into your step! In addition to providing helpful and innovative tips and tricks on what and how to eat to reach optimal health she will also demo a few healthy easy-to-make energy boosting snack recipes! Do not miss out!

Causes of fatigue:

- Cancer treatment in general
- Stress
- Anemia
- Lack of B vitamins
- Medication
- Sedentary lifestyle

Side effects of fatigue:

- Lack of sleep
- Loss of appetite
- Dehydration
- Mental fuzziness
- Coordination is off
- More accident prone
- Irritable

Every day foods to fuel your body and increase your energy

- Foods rich in iron – beans, leafy greens, lentils, tofu, tempeh, nuts, seeds, whole grains, dried fruits
- Foods rich in B vitamins – whole grains, fruits, nuts, herbs, nutritional yeast
- Foods rich in vitamin C – Red pepper, strawberries, citrus fruit, kiwi, Mango, raspberries, sweet potato, spinach
- Foods rich in electrolytes (calcium, potassium, chloride, magnesium, sodium) – chia seeds, coconut water, bananas, smoothies, homemade sports drinks (citrus juice, maple syrup, pinch of salt and mineral water)

Foods to avoid

- Foods rich in high simple carbohydrates – white processed sugar, brown sugar, cane sugar, etc.

Figure S3. Examples of NJSC-developed cancer nutrition resources.

Shown are the cover pages of NJSC-developed materials, including (A) *Eating for everyday energy: Fighting fatigue with food and lifestyle!*, (B) *Gilda's Nutrition Showcase: Foods to support your immune system!*, and (C) *Eating for everyday energy: Fighting fatigue with food and lifestyle!*

NJSC Menu: May 30 & June 1 2017

Soup: Creamy Corn Chowder with tempeh chorizo (recipe provided)

Salad: Mixed greens, nuts and seeds with an apple cider vinaigrette

Main: Kung pao chickpeas over sesame fried rice (recipe provided- millet was substituted for rice)

Dessert: Apple streusel cake with walnuts (recipe provided - strawberries were substituted for apples and hazelnuts for walnuts)

Pasta: Kids pasta (homemade marinara sauce over whole grain pasta)*

Figure S4. Example of the Not-Just-Supper Club Program's Menu.

*a pasta dish was included every week for selective children and those who couldn't tolerate super flavourful foods due to side effects of their treatments

Table S1. Crude and adjusted associations between participation at Not-Just-Supper Club and major protein foods (n=41).

Food group	Protein type	β (95% CI), unadjusted	*p*	β (95% CI), adjusted[a]	*p*[a]
**Nuts**	Plant	0.55 (0.20, 0.91)	0.003	0.49 (0.02, 0.96)	0.040
**Seeds**	Plant	0.26 (−0.19, 0.71)	0.246	0.30 (−0.22, 0.81)	0.245
**Pulses**	Plant	0.01 (−0.09, 0.11)	0.828	0.05 (−0.07, 0.16)	0.445
**Red meat**	Animal	0.00 (−0.08, 0.09)	0.937	0.02 (−0.07, 0.12)	0.648
**Dairy**	Animal	0.00 (−0.34, 0.34)	0.988	−0.04 (−0.36, 0.28)	0.809
**Fish**	Animal	−0.01 (−0.05, 0.04)	0.767	−0.02 (−0.08, 0.04)	0.554
**Eggs**	Animal	−0.02 (−0.07, 0.04)	0.585	−0.02 (−0.08, 0.04)	0.533
**Poultry**	Animal	−0.02 (−0.09, 0.06)	0.652	−0.02 (−0.10, 0.06)	0.613
**Soy foods**	Plant	−0.04 (−0.09, 0.01)	0.106	−0.04 (−0.09, 0.01)	0.105

Associations between duration of participation at the Not-Just-Supper Club (NJSC) and intake of animal- and plant-based protein food groups. β coefficients and 95% confidence intervals (CIs) were estimated using linear regression models for each additional year of participation at NJSC and food group intake (servings/day). *P*-values correspond to tests of the regression coefficients.
[a]Adjusted models include age (continuous), sex (female, male), ethnicity (Asian, Black, Other, White), and body mass index (kg/m²), and were among participants with complete covariate data (n=37).

Table S2. Association between time at Not-Just-Supper Club and nut intake by sex (n=41).

Sex	β (95% CI), unadjusted	β (95% CI), adjusted[a]	p[a]	*p* for interaction
**Females**	0.60 (0.21, 0.98)	0.56 (0.004, 1.12)	0.0486	0.415
**Males**	−0.37 (−1.04, 0.29)	−0.51 (−2.92, 1.90)	0.227	

Associations between duration of participation at the Not-Just-Supper Club (NJSC) and nut intake by sex. β coefficients and 95% confidence intervals (CIs) were estimated using multivariable linear regression models representing the change in nut intake (servings/day) per additional year of participation at NJSC. Sex-stratified analyses were exploratory. *P*-values correspond to tests of the regression coefficients. *P*-value for interaction corresponds to effect modification by sex assessed using a multiplicative interaction term between time at NJSC and sex.
[a]Adjusted models include age (continuous), ethnicity (Asian, Black, Other, White), and body mass index (kg/m²), and were restricted to participants with complete covariate data (females: n = 32; males: n = 5; total n = 37).

Table S3. Associations of soy intake and breast cancer among participants at Not-Just-Supper Club (n=41).

Cancer type	n	Consumers (%)	Mean soy intake (servings/day)	SD	*p*
Not breast cancer	31	96.8	0.17	0.22	
Breast cancer	10	100.0	0.33	0.43	0.30

P-value from Welch two-sample t-test comparing mean soy intake (servings/day) between groups. Analyses are exploratory and conducted post hoc.

Table S4. Servings of individual nut types and their correlations with time spent at the Not-Just-Supper Club (NJSC) (n = 41).

Nut type	Consumers, n (%)	Mean intake (serv/day), SD	Pearson r with time at NJSC	*p*
**Walnuts**	29 (70.7)	0.38 (0.62)	0.45	0.004
**Almonds**	38 (92.7)	0.62 (1.08)	0.23	0.17
**Pecans**	21 (51.2)	0.21 (0.47)	0.35	0.029
**Brazil nuts**	18 (43.9)	0.28 (0.61)	0.21	0.21
**Hazelnuts**	13 (31.7)	0.10 (0.24)	0.39	0.012
**Peanuts**	25 (61.0)	0.19 (0.28)	0.11	0.49

Consumers were defined as participants reporting intake of the specified nut at least once per month. Servings/day values are presented as mean (SD) serving per day. P-value is for correlation.

Table S5. Crude and adjusted associations between participation at Not-Just-Supper Club and nut types (n=41).

Nut subtype	β (95% CI), unadjusted	p	β (95% CI), adjusted	*p*
**Walnuts**	0.16 (0.05, 0.26)	0.004	0.11 (−0.03, 0.24)	0.106
**Almonds**	0.14 (−0.06, 0.34)	0.171	0.11 (−0.16, 0.37)	0.423
**Pecans**	0.09 (0.01, 0.18)	0.029	0.06 (−0.05, 0.18)	0.257
**Brazil nuts**	0.07 (−0.04, 0.19)	0.214	0.08 (−0.06, 0.22)	0.235
**Hazelnuts**	0.06 (0.01, 0.10)	0.012	0.06 (0.01, 0.10)	0.017
**Peanuts**	0.02 (−0.03, 0.07)	0.485	0.04 (−0.02, 0.11)	0.176

Associations between duration of participation at the Not-Just-Supper Club (NJSC) and intake of nut subtypes. β coefficients and 95% confidence intervals (CIs) were estimated using linear regression models for each additional year of participation at NJSC and nut intake (servings/day). Analyses of nut subtypes were exploratory and conducted post hoc.
P-values correspond to tests of the regression coefficients.
[a]Adjusted models include age (continuous), sex (female, male), ethnicity (Asian, Black, Other, White), and body mass index (kg/m^2), and were among participants with complete covariate data (n=37).
